# Maintaining Fluoroquinolone Class Efficacy: Review of Influencing Factors

**DOI:** 10.3201/eid0901.020277

**Published:** 2003-01

**Authors:** W. Michael Scheld

**Affiliations:** * University of Virginia, School of Medicine, Charlottesville, Virginia, USA

**Keywords:** Antibiotic resistance, levofloxacin, ciprofloxacin, moxifloxacin, ofloxacin, gatifloxacin, fluoroquinolones, Streptococcus pneumoniae, Pseudomonas aeruginosa, respiratory tract infections, urinary tract infections, perspective

## Abstract

Previous experience with antimicrobial resistance has emphasized the importance of appropriate stewardship of these pharmacotherapeutic agents. The introduction of fluoroquinolones provided potent new drugs directed primarily against gram-negative pathogens, while the newer members of this class demonstrate more activity against gram-positive species, including *Streptococcus pneumoniae.* Although these agents are clinically effective against a broad range of infectious agents, emergence of resistance and associated clinical failures have prompted reexamination of their use. Appropriate use revolves around two key objectives: 1) only prescribing antimicrobial therapy when it is beneficial and 2) using the agents(s) with optimal activity against the expected pathogens(s). Pharmacodynamic principles and properties can be applied to achieve the latter objective when prescribing agents belonging to the fluoroquinolone class. A focused approach emphasizing “correct-spectrum” coverage may reduce development of antimicrobial resistance and maintain class efficacy.

Development of resistance to antimicrobial agents and the emergence of multiresistant pathogens have generated worldwide concern in the medical community. Infections caused by resistant bacteria are associated with higher rates of hospitalization, greater length of hospital stay, and higher rates of illness and death (*1*,*2*). The estimated annual cost of treating infections caused by resistant bacteria in the United States is several billion dollars (*3*).

Antimicrobial resistance develops when bacteria are exposed to an antimicrobial agent, and selective pressure favors the growth of the resistant pathogen. To decrease selective pressure, antibacterial therapy should only be prescribed in patients with known or suspected bacterial infections. The risk for resistance can be further reduced by using an antimicrobial agent that has potent activity against the suspected pathogens at the dose and dosing frequency that maximizes its effectiveness.

Historically, several approaches to antibiotic prescribing have been employed to address antimicrobial resistance. One approach is to use a newer more potent antimicrobial in settings where resistance has emerged to an older agent. However, if newer agents are overused or used inappropriately, resistance will invariably develop to the newer drug. For example, since the late 1980s and early 1990s, ceftazidime, a third-generation cephalosporin, has been widely used against gram-negative pathogens, including *Pseudomonas*. However, indiscriminate use led to decreased activity against gram-negative infections and may have contributed to emergence of potent broad-spectrum β-lactamases among *Enterobacter*, *Citrobacter*, *Klebsiella*, and other gram-negative species (*4*–*7*). Another approach to combating resistance is to continue using older agents as first-line choices, in preference to newer, more potent drugs, in an effort to preserve the activity of the new drugs. The newer agents are reserved for infections caused by mutated multiresistant strains. However, as resistance continues to increase to the first-line agents, poor outcomes and secondary costs associated with clinical failures increase. By withholding the more potent agents for selected cases, these agents are increasingly compromised by the emergence of mutants selected by the less potent compounds.

An approach designed to reduce the rate at which antibiotic resistance develops is antibiotic cycling or rotation. It has been used with some success in intensive-care units (ICUs), where one class of agent has been predominantly used for a predefined period, usually 3 months, followed by use of another class for 3 months. Although not widely used, rotation has been used succesfully by Kollef et al. (*8*). A second approach is the use of combination therapy, whereby the additive or synergistic action of two or more drugs is exploited. Overall, resistance potential is theoretically minimized by this technique since these agents are typically from different antimicrobial classes, and different sites in the bacterial cell are targeted. Finally, a more focused approach of using the agents that demonstrate the best pharmacokinetic and pharmacodynamic profile against suspected pathogens might also reduce antimicrobial resistance. The objective of this approach is to predictably eradicate bacteria so that resistant clones are not selected.

The fluoroquinolone class of antimicrobial agents is being used empirically in an increasing number of patients because resistance has developed to the more traditional empiric agents. Fluoroquinolones are active against a wide range of multiresistant pathogens since their mode of action is against different molecular targets than other antimicrobial classes (*9*). Moreover, mechanisms of resistance to fluoroquinolones, apart from two unusual exceptions (*10**,**11*), are unlike almost all other class resistance mechanisms, being neither plasmid nor integron mediated.

We propose a strategy to preserve susceptibilities to this important antimicrobial class. Appropriate and targeted use of the fluoroquinolone class is discussed and analyzed within the context of in vitro, pharmacokinetic, and pharmacodynamic activity. The epidemiologic and clinical aspects of fluoroquinolone usage are outlined in an attempt to identify outcome-optimizing drug selection strategies. Finally, once fluoroquinolone therapy has been chosen, evidence-based strategies for how this antimicrobial class can be used to minimize development of drug resistance are discussed.

## Fluoroquinolone Differentiation: in vitro Perspectives

Individual members of the fluoroquinolone class demonstrate different spectra of activity and pharmacokinetic profiles. The first-generation fluoroquinolones (e.g., ciprofloxacin, ofloxacin, norfloxacin) are primarily active against gram-negative and some gram-positive organisms. The second-generation fluoroquinolone, levofloxacin, is the L-isomer of ofloxacin and demonstrates somewhat improved gram-positive activity. However, susceptibility data show levofloxacin to be less potent than ciprofloxacin against such gram-negative pathogens as *Pseudomonas aeruginosa* and certain enterobacteriaceae (*12*). The third-generation fluoroquinolones include moxifloxacin and gatifloxacin and have improved gram-positive, atypical, and anaerobic coverage compared with first- and second-generation fluoroquinolones. In particular, these newer representatives of the fluoroquinolone class manifest greater activity against *Streptococcus pneumoniae*, an important respiratory pathogen (*12*).

The relative activities of these fluoroquinolones, expressed as 90% MICs (MIC_90_s), are shown in Table 1. Ciprofloxacin is the most active fluoroquinolone against *P. aeruginosa;* typical MICs of ciprofloxacin are two- to eightfold lower than those of levofloxacin or newer quinolones such as moxifloxacin and gatifloxacin (*12*–*16*). Species of enterobacteriaceae also differ in their susceptibility to the quinolones (*12*). Ciprofloxacin is generally twofold more active against *Escherichia coli* and *Klebsiella pneumoniae* than levofloxacin and moxifloxacin (Table 1).

**Table 1:** Comparison of in vitro activity^a^ of four fluoroquinolones against a range of pathogens^b^

Fluoroquinolone	*E. coli*	*P. aeruginosa*	*K. pneumoniae*	*S. pneumoniae* ^c^	*S. aureus* ^d^	Ref.	
Ciprofloxacin	0.03	8	NR	4	0.5	12	
	0.125–0.5	0.25–4	0.25	1-2	0.5–1	13	
	0.016	8	0.06	4	0.5	14	
	0.016	2	0.25	2	0.5	15	
	0.25	4	0.06	2	1	16	
Levofloxacin	NR	32	NR	2	0.25	12	
	0.06–<0.5	0.5–>4	0.12–0.25	1–2	0.25	13	
	0.03	32	0.13	2	0.25	14	
	0.06	4	0.25	2	0.25	15	
	0.12	16	0.12	1	0.5	16	
Moxifloxacin	0.06	8	NR	0.25	0.06	12	
	0.06–1	8	0.12–0.25	0.06–0.25	0.12	13	
	0.008	32	0.13	0.25	0.06	14	
	0.06	8	0.5	0.25	0.06	15	
	0.5	8	0.5	0.25	0.12	16	
Gatifloxacin	NR	32	NR	1.0	0.125	12	
	0.06	>4	0.06–0.25	0.5	0.12	13	
	0.016	32	0.13	1	0.13	14	
	0.1	8	0.12	0.5	0.12	15	

^a^MIC_90_ reported. ^b^*E. coli, Escherichia coli; P. aeruginosa*, *Pseudomonas aeruginosa; K. pneumoniae, Klebsiella pneumoniae; S. pneumoniae, Streptococcus pneumoniae*; *S. aureus, Staphylococcus aureus;* NR, not reported. ^c^Penicillin-susceptible *S. pneumoniae,* except in the case of Reference 12, which did not specify. ^d^Methicillin-susceptible *S. aureus,* except in the case of Reference 12, which did not specify.

Conversely, ciprofloxacin (1.0–4.0 mg/L) and levofloxacin (1.0–2.0 mg/L) are not as active against *S. pneumoniae* as moxifloxacin (0.06–0.25 mg/L) or gatifloxacin (0.5–1.0 mg/L) (*12*–*16*). A recent survey conducted in the United States and Canada showed ciprofloxacin MIC_90_s of 2 mg/L against *S. pneumoniae* to be identical to those of levofloxacin but higher than those of the third-generation fluoroquinolone gatifloxacin (0.5 mg/L) (*17*). In addition to improved gram-positive activity, third-generation fluoroquinolones have improved activity against some anaerobic species compared to first- and second-generation fluoroquinolones. MIC_90_s for *Prevotella/Porphyromonas*, *Fusobacterium* species, and *Peptostreptococcus* species for levofloxacin are 1.0–8.0, 1.0–8.0, and 4.0 mg/L, as compared with moxifloxacin (0.5–4.0,0.125–4.0, and 0.5 mg/L), respectively (*18*). Activity of newer fluoroquinolones against a variety of atypical organisms is also improved. For example, for *Mycoplasma pneumoniae,* MICs are 1.0 mg/Land 0.5 mg/L for ciprofloxacin and levofloxacin, respectively, and 0.125 mg/L for both moxifloxacin and gatifloxacin (*19*).

Fluoroquinolone MIC_90_s will increase as resistant mutants invariably emerge, although the rate at which resistance develops largely depends on appropriate use. Patient-, institution-, and infection-specific therapeutic decisions require that antimicrobial susceptibilities be routinely tested and reported. Accurately assessing these MIC changes depends on the precision of the test used. The standard doubling dilution techniques used in most hospital microbiology laboratories may not be precise enough to identify minor susceptibility changes within a bacterial population (*20*). Utilizing the E-test method, which is sensitive enough to detect these subtle MIC changes (*21*), as follow-up for monitoring and controlling resistant strains isolated with increasing frequency in the clinical laboratory might be a practical solution, even though this approach requires greater resource and acquisition costs. Detecting and reporting these susceptibility changes are important since they can predict changes in the resistance potential of a pathogen (*22*). These data may be used to develop appropriate prescribing patterns to preserve antimicrobial activity.

Moreover, susceptibility data may not be accurate because surrogate methods, such as class-representative disk testing, are used in many institutions (*23*). Fuchs et al. (*24*) found that an accurate prediction of levofloxacin resistance could not be derived from use of ciprofloxacin or ofloxacin disk testing. This study showed that levofloxacin MICs were underestimated. Accordingly, the drug whose clinical use is being considered must be tested directly.

After observing three failures in patients treated with levofloxacin for pneumococcal infections, Davidson et al. conducted a survey in 2000 (*25*) and found that 86% of Canadian laboratories tested only nonfluoroquinolone antimicrobial agents for *S. pneumoniae* susceptibility. Given the growing resistance to traditional first-line agents and the increasing number of guidelines promoting quinolones as an alternative first-line choice in some patients (*26*–*28*), relevant testing should be routinely performed. Highlighting the need for fluoroquinolone susceptibility testing, Sahm et al. (*29*) noted a significant (p<0.005) increase in pneumococcal levofloxacin resistance in1997–1998 and 1998–1999 from 0.1% to 0.6%, although the incidence of *S. pneumoniae* resistance to fluoroquinolones remains low (<1%) in the United States (*30*,*31*).

## Resistance Selection in vitro: Mechanisms and Implications

Pathogenic bacteria employ a variety of strategies to persist and replicate under adverse conditions such as exposure to an antimicrobial agent. The efflux pump system is a mechanism that allows immediate survival of bacteria in the presence of an antimicrobial agent by actively expelling that agent across the cell membrane, thereby reducing the intracellular concentrations to sublethal levels. The pump’s action is dependent on the antimicrobial’s ability to bind to the bacterial efflux protein and be exported. Some fluoroquinolones, such as moxifloxacin and trovafloxacin, are not as affected by bacterial efflux mechanisms because of their bulky side-chain moiety at position 7, which hinders export (*32*).

Another resistance mechanism involves specific point mutations that reduce the binding of the antimicrobial agent to specific enzymatic sites by altering the target site. In this regard, fluoroquinolones bind to enzymes involved in DNA replication, including DNA gyrase and DNA topoisomerase IV. Specific mutations in the genes that code for these enzymes can result in decreased binding and activity of the fluoroquinolones (*33*). Different fluoroquinolones demonstrate stronger or weaker affinity to these enzyme-binding sites. First- and second-generation fluoroquinolones bind primarily to DNA gyrase or DNA topoisomerase IV, depending on the bacteria and the drug, whereas the third-generation fluoroquinolones generally bind strongly to both DNA gyrase and DNA topoisomerase IV. Thus, a single point mutation in DNA gyrase or DNA topoisomerase IV generally affects first- and second-generation fluoroquinolones to a greater extent than third-generation fluoroquinolones. Furthermore, the third-generation C-8 methoxyfluoroquinolones, moxifloxacin and gatifloxacin, appear to bind different molecular sites within these enzymes, thereby decreasing the cross-resistance between these agents and the older fluoroquinolones (*34*,*35*).

The rate at which resistance develops to an antimicrobial agent is a measure of the resistance potential of the agent and can be assessed in vitro. M’Zali et al. (*36*) compared the mutant selecting potential of ciprofloxacin and levofloxacin in *Pseudomonas aeruginosa*. In this study, clinical isolates of *P. aeruginosa* were repeatedly exposed to concentrations below the MICs for ciprofloxacin and levofloxacin. The fluoroquinolone-resistant strains emerged at a significantly increased rate with levofloxacin compared to ciprofloxacin (p<0.001). These findings were consistent with those of Gilbert et al. (*37*).

Likewise, Dalhoff et al. (*38*) compared the resistance selection potential of various fluoroquinolones in vitro after repeated overnight exposures to suboptimal concentrations of *S. pneumoniae*. In this study, the C-8-methoxyquinolones (moxifloxacin and gatifloxacin) showed a lower propensity to select resistant mutants compared with levofloxacin and ofloxacin.

Appropriate Fluoroquinolone Selection: Pharmacokinetic and Pharmacodynamic Considerations

Pharmacokinetic properties, including the concentration of drug in the serum over time (area under the curve [AUC]) and the peak serum concentration of the drug (Cmax), can be measured, and when considered in combination with in vitro activity, may be useful for predicting microbiologic success and clinical outcomes. In particular, the ratio of the Cmax to MIC or AUC to MIC (AUIC) can be predictive of drug efficacy, although which parameter is most predictive of clinical outcome is the subject of some disagreement. Generally, the higher the ratio, the better the outcome (*39*,*40*).

While fluoroquinolones are generally concentration-dependent bactericidal agents, differences in antibacterial activities exist among class members. Fluoroquinolones also differ in pharmacokinetic parameters, such as Cmax and AUC (*39*). These efficacy parameters, as they relate to *S. pneumoniae* and *P. aeruginosa,* for ciprofloxacin, levofloxacin, moxifloxacin, and gatifloxacin are shown in Table 2. Cmax/MIC and AUIC are highest for ciprofloxacin against *P. aeruginosa;* against *S. pneumoniae,* these values are highest with moxifloxacin.

**Table 2:** Comparison of pharmacokinetic and pharmacodynamic parameters for four fluoroquinolones and selected bacterial species^a^

				*Streptococcus pneumoniae*			*Pseudomonas aeruginosa*			
Fluoroquinolone	Dose (mg)	Cmax^a,b^ (mg/L)	AUC_24_^b^ (mg x h/L)	MIC^c^	Cmax:MIC	AUIC	MIC^c^	Cmax:MIC	AUIC	
Ciprofloxacin	500	3.0	28	2	1.5	14	4	0.75	7	
	750	3.6	32	2	1.8	16	4	0.9	8	
Levofloxacin	500	5.7	48	1	5.7	48	16	0.36	3	
Moxifloxacin	400	4.5	48	0.25	18	192	8	0.56	6	
Gatifloxacin	400	4.2	34	0.5	8.4	68	8	0.52	4.25	

^a^Cmax, peak serum concentration of the drug; AUC, area under the curve; MIC, minimal inhibitory concentration; AUIC, ratio of the AUC to MIC. ^b^References 41–44. ^c^Reference 16.

Although AUC/MIC and Cmax/MIC ratios are useful for predicting antimicrobial efficacy, they may not be as useful for predicting the potential for drug resistance to develop. In this regard, Thomas et al. (*45*) suggest that AUC/MIC should exceed 100 for gram-positive and gram-negative species to prevent resistance selection.

Alternatively, Zhao et al. (*46*) have hypothesized that the rate at which resistance develops to a fluoroquinolone is related to its MICs and mutant prevention concentrations. Studies involving a range of bacterial species suggest that the concentration to prevent mutant emergence in the clinical setting can be derived in vitro and is 2 to 4 times higher than the MIC for most fluoroquinolones (*46*); however, the clinical significance of these findings has not been clearly established. Derivation of the mutant prevention concentrations is a process involving spreading a high bacterial load onto a series of agar plates in which various concentrations of antimicrobial have been incorporated. The density of 10^10^ CFU/mL was selected to pinpoint frequency of mutation at levels of 10^-7^, 10^-8^, and 10^-9^, as well as to model the bacterial load at the site of infection. The inoculated plates are incubated overnight and the MIC of surviving colonies determined. This method has been applied to two species, *S. pneumoniae* and *P. aeruginosa,* for several fluoroquinolones (Table 3) (*47*,*48*).

**Table 3:** Mutant prevention concentrations (MPC)^a^ for various fluoroquinolones to *Streptococcus pneumoniae* and *Pseudomonas aeruginosa*^b^

Fluoroquinolone	Daily dose (mg)	Cmax^c^ (mg/L)	*P. aeruginosa* MPC (mg/L)	*S. pneumoniae* MPC (mg/L)
Ciprofloxacin	500 b.i.d.	3.0	2	NR^d^
	750 b.i.d.	3.6	2	NR
Levofloxacin	500 q.d.	5.7	8	8
Moxifloxacin	400 q.d.	4.5	NR	2
Gatifloxacin	400 q.d.	4.2	NR	4

^a^MPC values are derived from a study of approximately 100 isolates and are considered provisional. ^b^See references 47 and 48 for *S. pneumoniae* and *P. aeruginosa,* respectively. ^c^Cmax, peak serum concentration of the drug (41–44). ^d^NR, not reported.

Moxifloxacin exceeds the mutant prevention concentrations for *S. pneumoniae,* and ciprofloxacin exceeds the mutant prevention concentration for *P. aeruginosa* (both, 2 mg/L) by achieving maximum serum concentrations of 4.5 mg/L and 3.0 mg/L, respectively. These serum concentrations significantly exceed the mutant prevention concentrations; therefore, these agents are postulated to prevent mutant selection of *S. pneumoniae* and *P. aeruginosa,* respectively. Levofloxacin does not achieve mutant prevention concentrations of 8 mg/L in serum and thus may not inhibit mutant selection (*47*,*48*).

## Clinical Consequences of Inappropriate Use

Approval of ciprofloxacin in the United States in 1987 was accompanied by its rapid inclusion onto most hospital formularies. Initial use was predominantly for *P. aeruginosa* and other problematic gram-negative infections. However, after ofloxacin was introduced in 1992, some formularies substituted this drug for ciprofloxacin on the basis of cost alone. Similarly, levofloxacin was approved by the U.S. Food and Drug Administration in 1997–1998 for a broad range of infections and was added to formularies in an effort to reduce costs. The clinical consequences of these substitutions was not apparent at the time; however, the epidemiologic data soon emerged that reflected how varying levels of antimicrobial activity could make an impact on pathogen susceptibility and clinical outcomes.

Peterson and colleagues (*49*) noted decreases in *P. aeruginosa* susceptibilities to ciprofloxacin and ofloxacin of 21% and 23%, respectively, from 1992 to 1994. This decrease occurred after their medical center switched from ciprofloxacin to ofloxacin as the primary quinolone. In 1994, ciprofloxacin was reintroduced as the primary quinolone, and a 7% recovery in ciprofloxacin activity to *P. aeruginosa* was reported within 6 months.

Similarly, Rifenburg et al. (*50*) assessed the effect of fluoroquinolone usage on *P. aeruginosa* susceptibilities and collated data from 109 hospitals during 1993 to 1996. Greater use of ofloxacin was associated with lower *P. aeruginosa* susceptibilities. Bhavnani et al. (*51*) collected data from 145 hospitals and found a significant correlation between use of ofloxacin, but not ciprofloxacin, and decreasing *P. aeruginosa* susceptibilities. Additionally, the study suggested a deleterious effect of levofloxacin use on *P. aeruginosa* susceptibilities (*51*).

Introduction of levofloxacin in 1998 to replace ciprofloxacin in a tertiary-care university medical center resulted in a significant decrease in *P. aeruginosa* susceptibilities (from 74% to 57% in a 3-year period) and *E. coli* susceptibility to ciprofloxacin (from 99% to 89% in a 3-year period). Levofloxacin use rose from 91.2 to 272.8 defined daily dose (DDD)/1,000 patient days (199%) in the 3-year period (*52*). This volume of usage exceeds that of 50 DDD/1,000 patients, a threshold suggested by Austin et al. (*53*) as a predictive driver in selecting for antimicrobial resistance during a 2-year period. Zambrano et al. (*54*), at the same institution, recently reported a significant correlation between increased levofloxacin use and declining fluoroquinolone susceptibilities among ICU isolates of *K. pneumoniae* (96% to 79% [p<0.008]) and *P. aeruginosa* (82% to 67% [p<0.01]).

Similarly, another group reported (*55*) that after levofloxacin was added to the formulary, levofloxacin use as a proportion of total fluoroquinolone use increased from <2% to >22% over a 6-month period (from 3rd quarter 1999 to 1st quarter 2000). During the period of 1st quarter 1998 to 2nd quarter 2000, the susceptibility of *P. aeruginosa* to ciprofloxacin decreased by 11% (82% to 71%). The use of parenteral antipseudomonal agents such as gentamicin, imipenem, ceftazidime, and piperacillin/tazobactam increased concurrently, suggesting that physicians began using non-fluoroquinolone combination therapy when treating serious gram-negative infections. Furthermore, the antimicrobial cost reductions anticipated from switching to a less expensive fluoroquinolone on formulary were not realized. In 3rd quarter 2000, levofloxacin was replaced with ciprofloxacin as the main gram-negative fluoroquinolone, a substitution associated with a subsequent 6% increase in ciprofloxacin activity against *P. aeruginosa* during the next year.

Because the ICU has been a focal point of antimicrobial resistance, the Centers for Disease Control and Prevention initiated Project ICARE in 1996 (*56*). Specific data regarding fluoroquinolone use and fluoroquinolone susceptibility among *P. aeruginosa* isolates were presented for the period 1996–1999 by Hill et al. (*57*). No correlation was found between prevalence of quinolone resistance and total use of ciprofloxacin/ofloxacin. However, significant associations were found between fluoroquinolone resistance and combined use of ciprofloxacin, ofloxacin, and levofloxacin (p<0.019) and by use of levofloxacin alone (p<0.006) (*57*).

Likewise, recent studies suggest that using a less potent fluoroquinolone against *S. pneumoniae* for treating community and hospital respiratory tract infections may be affecting the activity of all fluoroquinolones against this respiratory pathogen (*58*–*60*). Fluoroquinolone resistance in *S. pneumoniae* has been reported, most notably from Hong Kong (58). A 1998 study of 181 *S. pneumoniae* isolates showed resistance to ciprofloxacin in 12.1%, to levofloxacin in 5.5%, and to trovafloxacin in 2.2%. By early 2000, levofloxacin resistance had increased to include 13.3% of all *S. pneumoniae* and 27.3% among penicillin-resistant strains (*59*). These strains also demonstrated elevated MICs to newer class members such as gatifloxacin (12.8%) and moxifloxacin (12.2%) (the latter occurring exclusively in highly penicillin-resistant strains). Additionally, fluoroquinolone resistance appears to be emerging in other countries such as Canada, where resistance rates have increased from 0% in 1993 to 1.7% in 1997/1998 combined (*60*).

## Clinical Consequences of Inappropriate Fluoroquinolone Use

Inappropriate use of antimicrobial agents has been associated with adverse consequences, including therapeutic failure, development of resistance, and increased health-care costs. One example of a mismatch between pharmacodynamics and clinical infection was in the use of ciprofloxacin for community-acquired pneumonia. The pharmacodynamics of the dose typically prescribed in these cases (ciprofloxacin 250 mg b.i.d.) are inappropriate for treating pneumococcal pneumonia, especially in seriously ill patients (*41*). By 1994, approximately 15 cases of *S. pneumoniae* infections that did not respond to ciprofloxacin had been reported, primarily in seriously ill patients and associated with contraindicated medications and other important medical issues (*61*). These events prompted the U.S. Food and Drug Administration to modify the package insert to warn against empiric use of ciprofloxacin for respiratory infections in which *S. pneumoniae* would be a primary pathogen. Consequently, ciprofloxacin has been used less frequently in these types of infections.

By contrast, >50% of levofloxacin use has been for the treatment of respiratory infections. Since 1999, at least 20 case reports of pulmonary infections that did not respond to levofloxacin therapy have been published (*25**,**62**–**69*). Three of the patients died due to fulminant pneumococcal infections that were unresponsive to levofloxacin therapy at approved dosage (*25*,*62*,*69*). Very few of these cases were in immunosuppressed patients. Reports of pneumococcal failures on the standard dosage of levofloxacin, 500 mg every 24 h, have also been described in two clinical trials, one in a patient with acute exacerbation of chronic bronchitis and the other in a patient with community-acquired pneumonia (*70*,*71*) (Table 4). In some of the 21 case reports, the treatment failed, and the pathogen developed levofloxacin resistance during therapy.

**Table 4:** Clinical failures of *Streptococcus pneumoniae* infection with levofloxacin^a^

				Risk factors				
No. of cases	Age	Indication		Coexisting conditions	Prior FQ use	Yr	Ref.	Country
1^b^	58	Meningitis		HIV, splenectomy	NR	1999	62	USA
3	NR	RTI			Yes	1999	63	USA
1	63	CAP		COPD	No	1999	64	USA
1	50	CAP		COPD	No	2000	65	USA
1	84	CAP		COPD	Yes-Levo	2000	65	USA
1	53	HAP		none	No	2001	66	USA
7	39-83 (avg. 63)	4 CAP 3 AECB		COPD (5)	5/7 (4-Lev, 1-Mox)	2001	67	USA
1	37	CAP		none	No	1999	25	Canada
1^b^	66	CAP		COPD	Yes-Cip + Lev	1999	25	Canada
1	80	AECB/CAP		COPD,	Yes-Cip	2001	25	Canada
1	64	CAP		none	No	2000	25	Canada
1	50	CAP		COPD	Yes-Lev	2001	68	USA
1^b^	79	CAP		none	NO	1999	69	USA
21								
Clinical trials								
13 (7 on 500 mg)	NR		AECB	COPD	No		70	Neth.
4	NR		CAP	NR	No		71	USA
24 (11)								
Epidemiologic studies								
16^c^	-		LRTI	COPD	Yes-Cip	1995-96	72	Canada
27^d^	-		LRTI	COPD (17)	Yes-Lev	1998-99	73	HK
43								
Total	88 (74 on 500 mg) clinical/bacteriologic failures							

^a^FQ, fluoroquinolone; NR, not reported; RTI, respiratory tract infection; CAP, community-acquired pneumonia; COPD, chronic obstructive pulmonary disease; Lev, levofloxacin; Mox, moxifloxacin; HAP, hospital-acquired pneumonia; AECB, acute exacerbation of chronic bronchitis; LRTI, lower respiratory tract infection; Cip, ciprofloxacin; Neth, the Netherlands; HK, Hong Kong. ^b^Death. ^c^3 deaths. ^d^4 deaths.

Davidson et al. (*25*) recently published details of four cases of pneumococcal pneumonia in which levofloxacin therapy failed. Two of the patients had no history of prior fluoroquinolone use and were levofloxacin susceptible beginning therapy, but their *S. pneumoniae* isolates were levofloxacin-resistant after therapy. These resultant mutants exhibited increased MICs to the newer fluoroquinolones moxifloxacin and gatifloxacin as well, thus decreasing those agents’ potential effectiveness. Both Weiss et al. (*72*) and Ho et al. (*73*) demonstrated clear risk factors (Table 5) associated with the development of fluoroquinolone resistance, including prior exposure of the patient to first- or second-generation fluoroquinolones (i.e., ciprofloxacin, levofloxacin, and ofloxacin) and history of chronic obstructive pulmonary disease.

**Table 5:** Risk factors for infection or colonization with fluoroquinolone-resistant *Streptococcus pneumoniae*^a^

Factor	Case patients (n=27)	Control patients (n=54)	Odds ratio (95% CI)	p value	
Age (yr)^b^	72.5 (62.3–78.3)	75 (70–85)	–	0.01	
Nursing home residence	14 (52%)	7 (13%)	7.2 (2.4 to 21.6)	<0.001	
COPD	17 (63%)^c^	12 (22%)	5.9 (2.2 to 16.3)	0.001	
Nosocomial origin	18 (66%)	14 (26%)	5.7 (2.1 to 15.6)	0.001	
Interval from day of admission to isolation of LRSP (days)^b^	7 (1–20)	1 (1–3)	-	<0.001	
No. of prior admissions^b^	4 (2–7)	1 (0–3)	-	<0.001	
Recent hospitalization	16 (59%)	13 (24%)	4.6 (1.7 to 12.3)	0.003	
Multiple hospitalization	15 (56%)	12 (22%)	4.4 (1.6 to 11.8)	0.004	
Previous exposure to antimicrobial agents^d^					
Fluoroquinolones	8 (30%)/14 (52%)	0 (0%)/5 (9%)	―/10.6 (3.2 to 34.7)	<0.001/<0.001	
β-lactam antibiotics	24 (89%)/25 (93%)	20 (37%)/32 (59%):	14.7 (3.9 to 55.4)/8.6 (1.8 to 40)	<0.001/0.006	

^a^CI, confidence interval; COPD, chronic obstructive pulmonary disease; LRSP, levofloxacin-resistant *S. pneumoniae* (73). ^b^Median (interquartile range). ^c^Colonization in 3 patients. ^d^Exposure to antimicrobial therapy during the 6 weeks prior to hospitalization/12 months before hospitalization.

## Conclusions

The fluoroquinolone class of antimicrobial agents is being increasingly used empirically as resistance has developed to the more traditional antimicrobial agents. Guidelines now recommend fluoroquinolones as first-line empiric therapy for urinary tract infections in regions were trimethoprim/sulfamethoxazole resistance is >10% to 20% (*28*), and fluoroquinolones are recommended as alternative empiric regimens in some patients with community-acquired pneumonia (*26*,*27*). Though increased use of these agents would be expected to lead to increased resistance, a targeted approach to fluoroquinolone prescribing, emphasizing their appropriate use, may reduce development of antimicrobial resistance and maintain class efficacy.

Evidence is mounting that suggests a link between inappropriate fluoroquinolone use, development of antimicrobial resistance against the entire fluoroquinolone class, and clinical failure. To maintain the activity of the fluoroquinolone class, clinicians need to implement an evidence-based approach to antimicrobial selection, particularly a strategy in which the most pharmacodynamically potent fluoroquinolone is matched, on an empiric basis when required, to anticipated bacterial pathogens.

Three major factors are associated with increasing resistance to fluoroquinolones (*74*): 1) underdosing, i.e., use of a marginally potent agent whose MIC is barely reached in serum or infected tissues; 2) overuse of agents known to encourage resistant mutants; and 3) the inability to readily detect and respond to changes in antimicrobial susceptibilities. Traditional reporting of susceptibility data may be misleading and may not readily identify initial changes in resistance patterns or differences between agents of the same class.

To preserve fluoroquinolone activity, the activity of these agents must be continually assessed, and these agents must be used appropriately. The individual attributes of a given drug should be matched with the likely pathogen at specific infective sites. Expecting a single fluoroquinolone to be suitable for all infections is unreasonable, and excessive use of any single fluoroquinolone for all indications will lead to resistance that will adversely affect the entire class.

Given the defined strategy of selecting the agent with the best pharmacokinetic and pharmacodynamic profile against the known or suspected pathogen, an appropriate therapeutic choice for most serious infections, such as nosocomial pneumonia in which *P. aeruginosa* is a known or suspected pathogen, would currently include ciprofloxacin in combination with an antipseudomonal β-lactam or an aminoglycoside antibiotic. This recommendation is based on the lower MIC_90_ and mutant prevention concentrations for this fluoroquinolone against *P. aeruginosa* and higher Cmax/MIC and AUC/MIC ratios compared to other members of the class. Likewise, for most other gram-negative infections of the skin and urinary tract, including *P. aeruginosa* infections, ciprofloxacin monotherapy is appropriate.

Ciprofloxacin, levofloxacin, and gatifloxain all achieve high concentrations in urine; thus, they would all be appropriate choices for treating urinary tract infections in the community. Ciprofloxacin would be the most appropriate therapy in cases where *P. aeruginosa* is a known or suspected pathogen. For other gram-negative infections, levofloxacin or gatifloxacin should be prescribed in appropriate doses to surpass the mutant prevention concentrations at the infection site.

For infections in which *S. pneumoniae* is anticipated to be the most likely pathogen (e.g., community-acquired pneumonia), moxifloxacin, which currently has the best antipneumococcal pharmacodynamic activity and the lowest mutant prevention concentrations against this organism, would represent a prudent therapeutic choice. By contrast, levofloxacin MIC_90_s against *S. pneumoniae* are significantly higher than those of moxifloxacin and gatifloxacin. The AUC/MICs and Cmax/MICs are also lower for levofloxacin against *S. pneumoniae,* and serum concentrations of a standard dose of levofloxacin for community-acquired pneumonia do not reach the mutant prevention concentrations for *S. pneumoniae*. For these reasons, levofloxacin may not be the best choice for infections caused by *S. pneumoniae*. Furthermore, recent reports of levofloxacin failures in cases of community-acquired pneumonia caused by *S. pneumoniae* evoke concern.

The targeted strategy proposed in this review is being implemented in a variety of institutions since the introduction of the third-generation fluoroquinolones. Documenting the effect of this approach on hospital susceptibilities over time will be important. Additionally, susceptiblities in these hospitals need to be compared to those in hospitals that use a single fluoroquinolone more broadly.
